# Comparison of Diagnostic Methods in Patients with Squamous Intraepithelial Lesion in Women Infected with Multiple High-Risk Human Papillomaviruses

**DOI:** 10.30699/ijp.2024.2036312.3330

**Published:** 2025-01-10

**Authors:** Ensiyeh Bahadoran, Babak Rahmani, Esfandiyar Nazari, Aida Hosseinnezhad, Fatemeh Samieerad

**Affiliations:** 1 *Cellular and Molecular Research Center, Institute for Prevention of Non-Communicable Diseases, Qazvin University of Medical Sciences, Qazvin, Iran*; 2 *Department of Molecular Medicine, Faculty of Medical School, Qazvin University of Medical Sciences, Qazvin, Iran*; 3 *School of Medicine, Qazvin University of Medical Sciences, Qazvin, Iran*; 4 *Department of Pathobiology, School of Medicine,* *Qazvin University of Medical Sciences, Qazviin, Iran*

**Keywords:** Cervical cancer, Colposcopy, Diagnostic methods, Histopathology, HPV screening, Pap smear, Squamous intraepithelial lesions

## Abstract

**Background & Objective:**

Human papillomavirus (HPV) is a major cause of cervical cancer and mortality, particularly in low-income countries. Late diagnosis of cervical cancer often leads to advanced-stage disease, resulting in poorer prognosis and higher mortality, which underscores the critical need for effective early screening methods. Pap smears, colposcopy, and HPV testing are essential for early detection. This study addresses a gap in the literature by comparing the effectiveness of different diagnostic methods specifically in women with multiple high-risk HPV (hrHPV) infections—a population for which limited comparative data exist.

**Methods:**

This prospective cross-sectional study, approved by the Ethical Committee of Qazvin University (IR.QUMS.REC.1400.143), was conducted at Kowsar Hospital from 2022 to 2023 and included 608 patients infected with multiple hrHPV subtypes. Inclusion criteria required hrHPV confirmation by PCR genotyping and excluded patients without colposcopy and biopsy or with a history of cervical dysplasia. Participants underwent Pap smear cytology, colposcopy, and biopsy, with the biopsy specimens examined by a gynecologic pathologist. Statistical analyses included chi-square tests for qualitative variables and t-tests or ANOVA for quantitative variables, with a significance level set at P<0.05.

**Results:**

The average age of the patients was 38.54 years, with the majority aged 30–39 years (43.6%). Common symptoms included vaginal discharge (36.5%) and postcoital bleeding (34.9%). Pap smear results showed ASCUS in 43.6% of cases and LSIL in 39.1%. Colposcopy revealed acetowhite lesions in 45.6% and abnormal vascular patterns in 25.5%. Histopathology indicated that 72.4% of patients had CIN I. Smoking was significantly correlated with colposcopic findings (P=0.044). Colposcopy demonstrated the highest sensitivity (69.1%) for detecting cervical lesions, while cytology had the highest specificity (63.1%).

**Conclusion:**

Pap smear findings provide adequate diagnostic accuracy for hrHPV patients, but colposcopy offers superior sensitivity for detecting cervical lesions. Combining both methods is recommended to improve diagnosis.

## Introduction

Cervical cancer is the fourth most common malignancy among women after breast, colorectal, and lung cancers(1). In developing countries, cervical cancer is the primary cause of death from cancer among women, and over 85% of cervical cancer deaths occur in middle- and low-income countries (2,3). Persistent high-risk HPV (hrHPV) infection accounts for > 90% of cervical cancer cases and is the most prevalent HPV-related illness (4). More than 150 different varieties of HPV are categorized as high-risk (e.g. HPV types 16, 18, 31, 33, 35, 39, 45, 51, 52, 56, 58, 59, 68, 69, 82) and low-risk (e.g. HPV types 6, 8, 11, 40, 42, 43, 44, 54, 61, 72) based on their oncogenicity (5). HPV types 16 and 18 are responsible for nearly 70% of cervical cancer cases (6). The persistence of hrHPV and its association with cervical cancer is influenced by a combination of host lifestyle factors, including smoking, alcohol use, multiple sexual partners, STI co-infections, and contraceptive use (7).

Despite Iran's low cervical cancer incidence rate of 2.5 per 100,000 population, the mortality rate remains alarmingly high at 42%, primarily due to the late-stage diagnosis of many cases. Limited access to cervical cancer screening and significant disparities in healthcare utilization further exacerbate this issue. According to recent data, only 52.1% of women aged 30-59 have undergone screening, with participation rates varying dramatically across provinces, from 7.6% in Sistan and Baluchestan to 61.2% in Isfahan. Socio-economic factors such as illiteracy, lack of insurance coverage, and cultural barriers contribute to this low participation, leading to a higher likelihood of late-stage diagnosis and poorer outcomes (8,9).

However, the effect of multiple HPV infections on cervical disease remains controversial. Studies have shown that coinfection with multiple HPV types is a significant risk factor for the development and progression of cervical dysplasia. Moreover, HPV infections have a direct relationship with the severity of cervical intraepithelial neoplasia, and it appears that infections act synergistically in cervical cancer (10–13). On the other hand, alternative studies have shown that co-infection with multiple HPV types occurs at random and has no synergistic or additive effect on cervical disease progression or increased risk compared to a single HPV infection (14,15).

Pap smear, colposcopy, and cervical biopsy are common screening tests. Cervical cancer screening has the potential to effectively lower the incidence and mortality of cervical cancer, owing to the extended time lag between HPV infection and the development of cervical cancer (16). The primary screening test for the early diagnosis of invasive cervical cancer and precancerous cervical intraepithelial neoplasia is the Pap smear test, which has been effective in reducing female mortality rates (17, 18). Colposcopy is another diagnostic procedure that uses a magnified view to examine the cervix, as well as tissue samples from the urethra and vulva, and is performed in response to an abnormal cervical cancer screening test (2, 19). The definitive diagnosis of cervical intraepithelial lesions relies on histopathological examination of cervical lesion biopsies, which is the gold standard for accurate assessment (20). Since HPV is present in over 90% of cervical carcinoma cases, studies have indicated that HPV detection is superior to cytology for identifying severe cervical lesions. Supporting this, a large-scale study in Italy showed that HPV testing was more effective than traditional cytology in preventing invasive cervical carcinoma (21).

Given the high prevalence of cervical cancer in hrHPV patients, the significant expenditure on cancer treatment, and the high mortality rates—especially in developing countries—epidemiological studies are vital for disease control. Comparing various screening methods in patients infected with multiple hrHPVs helps to identify their strengths and weaknesses, allowing for improvements in efficiency. This comparison also highlights the need for additional tests, leading to the selection of the most effective methods and reducing the reliance on aggressive and expensive procedures. This study aims to address the existing gaps in research by comparing diagnostic methods for squamous intraepithelial lesions in women infected with multiple high-risk HPV strains, with the goal of identifying the most sensitive and specific approach for early detection and improved outcomes.

## Material and Methods

This cross-sectional prospective analytical descriptive study was conducted at Kowsar Hospital, affiliated with the Qazvin University of Medical Sciences, between 2022-2023. The study procedures were approved by the Ethics Committee of the Qazvin University of Medical Sciences, Qazvin, Iran (IR.QUMS.REC.1400.143), and written consent was obtained from all participants. In total, 608 patients who tested positive for multiple hrHPV subtypes were included in our evaluation. The inclusion criteria were all women infected with multiple hrHPV based on genotyping studies by PCR on cervical cytology fluid samples referred to Kowsar Hospital. Patients who did not undergo colposcopy and biopsy or who had a history of cervical dysplasia were excluded from the study. In cases of intraepithelial lesions in the pap smear and infection with multiple hrHPV in genotypic results, the pap smear cytology results were recorded in a checklist, and the patient was a candidate for colposcopy. A biopsy was performed on the suspicious and problematic areas during the examination. Biopsies were examined by an expert gynecology pathologist and reported according to the latest guidelines in this field.

All demographic, clinical, pap smear cytology, colposcopy, and histopathological information of women infected with multiple hrHPV were simultaneously recorded in a checklist. The first part of the checklist included demographic information, such as age, age at marriage, number of pregnancies, and smoking status. The clinical information included vaginal discharge, vaginal bleeding after intercourse, abnormal vaginal bleeding, and spotting. In this study, the conventional pap smear was used and the reporting of pap smear results was based on the latest version of Bethesda (21) and was reported as the degree of inflammation (mild, moderate, severe), HSIL, LSIL, ASC-H, ASCUS, LSIL+HSIL and normal. Colposcopy information included acetowhite, abnormal vascular pattern, abnormal cervix, ulcer, and other normal findings. Histopathology information includes cervicitis, CIN I, CIN I-CIN II, CIN II, CIN II-CIN III, and CIN III 

The data collected were analyzed using SPSS version 22 (SPSS Inc., Chicago, Ill., USA). In addition, the relationships between qualitative variables were analyzed using a chi-square test, quantitative variables were analyzed using a correlation test, and qualitative and quantitative variables were analyzed using a t-test or ANOVA. The sensitivity, specificity, and positive and negative predictive values of different diagnostic methods were calculated. The significance level for the interpretation of the results was 0.05. 

## Results

Demographic characteristics of women infected with multiple hrHPV viruses referring to Kowsar Hospital, including age, age at marriage, smoking, and number of pregnancies, are shown in Table 1. Based on these results, the age range of the patients was 20-75, with an average age of 38.54±9.3 years. The age range and average age at marriage were 12-42 and 22.88±3.93 years, respectively. Moreover, the average number of pregnancies of the patients was equal to 2.26±1.27 pregnancies. In addition, 4.1% of patients were smokers (Table 1).

**Table 1:** Demographic information of hrHPV patients

Demographic		Number	Percentage
Age	20-29	92	15.1
	30-39	265	43.6
	40-49	177	29.1
	50-59	58	9.5
	60<	16	2.6
Marriage year	<20	171	28.1
	21-25	306	50.3
	26-30	104	17.1
	31-35	24	3.9
	36<	3	0.5
Gravidia	0	32	5.3
	1	123	20.2
	2	224	36.8
	3	157	25.8
	4	48	7.9
	5	13	2.1
	6	7	1.2
	7	2	0.3
	10	1	0.2
	11	1	0.2
Smoking	Yes	25	4.1
	No	583	95.9

Absolute and relative frequencies of clinical symptoms, pap smear, inflammation, colposcopy, histopathology, and PCR findings in patients have been evaluated. Vaginal discharge (36.5%) and bleeding after intercourse (34.9%) were the most common symptoms observed. ASCUS was observed in 43.6% of cases, LSIL in 39.1%, HSIL in 3.6%, and ASC-H in 1.3% based on pap smear results. Mild inflammation was observed in 40.5% of patients. Colposcopy revealed acetowhite lesions in 45.6% and abnormal vascular patterns in 25.5% of the patients. Histopathology indicated that 72.4% had CIN I. 82.9% of the patients had infections with multiple high-risk HPV (HrHPV) strains, while 17.1% had infections with HrHPV strains along with low-risk HPV (lrHPV) types (Table 2). 

Sensitivity, specificity, positive predictive value (PPV), and negative predictive value (NPV) of colposcopy, clinical data, and cytology for the diagnosis of cervical lesions have been examined. Colposcopy had the the highest sensitivity (69.1%). Clinical data showed an intermediate sensitivity (61.5%), with the lowest specificity (33.7%) and NPV (30.3%). Cytology demonstrated the highest specificity (63.1%) and PPV (76.2%), but the lowest sensitivity (47.9%) (Table 3).

The results showed a significant correlation between pap smear cytology, colposcopy findings, and histopathological results (*P*=0.001). The highest percentage of LSIL (202 cases, 45.9%) in the pap smear was related to CIN I, as reported by histopathology. In addition, in the correlation between colposcopy and histopathological findings, most acetowhite changes were related to CIN1 (260 cases and 59.6%) (Table 4). 

The relationship between demographic factors and diagnostic methods in hrHPV patients have been evaluated. While most associations are not significant, smoking shows a potentially significant association with colposcopy (*P*=0.044). Additionally, the relationship between age and pathology yielded the next lowest p-value (*P*=0.079), though it did not reach statistical significance. Other variables like age, marriage year, and gravity display no meaningful associations across clinical findings, cytology, colposcopy, or histopathology. Overall, smoking seems to be the most relevant factor linked to diagnostic outcomes, while other factors do not appear to have a strong impact (Table 5).

**Table 2:** Absolute and relative frequency of findings from various diagnostic methods among hrHPV patients

Variable		Number	Percent
Clinical	Vaginal discharge	222	36.5
	Vaginal bleeding after intercourse	212	34.9
	Abnormal vaginal bleeding	86	14.1
	Spotting	88	14.5
Pap smear			
Squamous cell abnormality	ASCUS	265	43.6
	ACH-H	8	1.3
	LSIL	238	39.1
	HSIL	22	3.6
	LSIL-HSIL	8	1.3
	None	67	11.0
Inflammation	Mild	246	40.5
	Moderate	161	26.5
	Severe	51	8.4
	None	150	24.7
Colposcopy	Normal	129	21.2
	Acetowhite	277	45.6
	Abnormal Vascular Pattern	155	25.5
	Cervical abnormality	43	7.1
	Ulcer	4	.7
Histopathology	Cervicitis	130	21.4
	CIN I	440	72.4
	CIN II	14	2.3
	CIN III	8	1.3
	CIN I-CIN II	10	1.6
	CIN II-CIN III	6	1.0
PCR findings	Multiple hrHPV	504	82.9
	Multiple hrHPV and lrHPV	104	17.1

**Table 3:** Sensitivity, specificity, positive and negative predictive values of colposcopy, clinical data, and cytology compared with histopathology in hrHPV patients

	Sensitivity	Specificity	Positive predictive value	Negative predictive value
Colposcopy	69.1	42.5	74.9	40.1
Clinical Data	61.5	33.7	72.4	30.3
Cytology	47.9	63.1	76.2	35.2

**Table 4:** The relationship between histopathologic findings with cytology, colposcopy findings, and clinical symptoms among hrHPV patients

		Histopathologic findings						P-value
		Cervicitis	CIN I	CIN II	CIN III	CIN I-CIN II	CIN II-CIN III	
Clinical findings	vaginal discharge	33	173	6	4	4	2	0.486
	Vaginal bleeding after intercourse	50	149	4	1	4	4	
	Abnormal vaginal bleeding	28	55	1	1	1	0	
	spotting	19	63	3	2	1	0	
Cytology findings	ASCUS	63	202	0	0	0	0	0.000
	LSIL	0	236	0	0	2	0	
	HSIL	0	0	10	7	3	2	
	others	0	2	4	1	5	4	
Colposcopy findings	Normal	124	4	1	0	0	0	0.000
	Acetowhite	6	260	4	3	3	1	
	Abnormal Vascular Pattern	0	138	8	3	3	3	
	Cervical abnormality	0	34	1	2	4	2	

**Table 5:** Relationship between demographic variables and various diagnostic methods in hrHPV patients

		Clinical findings		Cytology		Colposcopy		Pathology	
		Vaginal Discharge	Others	ASUS	Others	Normal	Abnormal	Cervicitis	CIN(I- III)
Age	20-29 30-39 40-49 50-59 =>60	39 92 60 26 5	53 173 117 42 11	36 114 78 29 8	56 151 99 29 8	27 57 31 10 4	65 208 146 48 12	27 57 30 12 4	65 208 147 46 12
	P value	0.461		0.138		0.092		0.079	
Marriage year	<20 21-25 26-30 31-35 >36	56 111 45 9 1	115 195 59 15 2	79 129 45 10 2	92 177 59 14 1	39 71 15 4 0	132 235 89 20 3	40 72 14 4 0	131 234 90 20 3
	P-value	0.235		0.393		0.305		0.500	
Smoking	Yes No	9 213	16 370	12 253	13 330	4 125	21 458	5 125	20 458
	P-value	0.401		0.425		0.044		0.620	
Gravity	0 1 2 3 4 5 6 7 10 11	11 54 80 51 17 5 4 0 0 0	21 69 144 106 31 8 3 2 1 1	12 58 96 65 23 4 5 1 0 1	20 65 128 92 25 9 2 1 1 0	6 33 42 32 7 7 2 0 0 0	26 90 182 125 41 6 5 2 1 1	6 33 41 34 7 7 2 0 0 0	26 90 183 123 41 6 5 2 1 1
	P-value	0.372		0.489		0.577		0.279	

## Discussion

The present study examined 608 women infected with multiple hrHPVs based on genotypic studies of cervical cytology liquid samples referred to Kowsar Qazvin Hospital.

According to the findings, the average age of the patients with multiple hrHPV was 38.54 ± 9.3 years, which is incompatible with other studies (22,23). Coupe et al. demonstrated that at the age of 22 years, the prevalence of hrHPV peaked at 24%, and the prevalence of hrHPV among women aged 29 to 61 years significantly decreased as age increased for all types of hrHPV (24). In the current study, patients aged 30–39 years had the highest frequency (>43%). Similarly, in the Iranian population, the highest rate of HPV-16 infection occurred in patients aged 30–44 (25), as most women in the study population belonged to this age group.

Moreover, the average married age of the patients was 22.88 ± 3.93 years and the average number of pregnancies was 2.26 ± 1.27. The married age of the studied patients was lower than the national average (26). In addition, the average number of pregnancies in the studied patients was higher than the national average (27). The lower married age and higher number of pregnancies likely increase the duration of sexual activity and exposure to HPV, factors that have been linked to an elevated risk of HPV infection and cervical disease (28,29). Four point one percent of patients smoked, which is almost equal to the average smoking rate of women in the country (4.44%) (30).

According to the clinical findings, vaginal discharge and bleeding during intercourse were the most frequent symptoms (36.5% and 34.9%, respectively). The most common clinical findings in other studies of HPV-infected patients were vaginal discharge, abnormal uterine bleeding, pelvic inflammatory disease, and lower abdominal pain (31,32). Premalignant and inflammatory lesions of the cervix explain this finding.

Based on the cytology findings, ASCUS (43.6%) and LSIL (39.1%) were the most frequent, and no significant relationship was observed between the cytology findings and the demographic characteristics of the patients. Owing to ambiguous cellular changes, inflammatory and non-malignant lesions may resemble squamous intraepithelial lesions. Therefore, an ASCUS diagnosis is more common than other squamous intraepithelial lesion diagnoses to avoid missing probable premalignant cellular alterations (20). In a study conducted by Cetin et al. on 279 HPV-positive patients, reference smear results identified 48.8% and 23.9% of patients as ASCUS and LSIL, respectively (33). Elfström et al. showed that during a 14-year monitoring period in a randomized HPV screening experiment, hrHPV strains played a significant role in the development of low-grade squamous intraepithelial lesions (LSILs). Moreover, the risks associated with HPV for atypical squamous cells of ASCUS/LSIL are notably influenced by time factors (34). Schlecht et al. demonstrated that ASCUS lesions positive for hrHPV have an 18% chance of progressing to a higher-grade lesion within 18 months, whereas LSIL lesions have a 12% chance of progressing (35). The treatment of ASC-US cytology includes HPV testing, urgent colposcopy with or without biopsy, and a repeat cytology test every four to six months (36). Our study’s ASCUS rate of 43.6% is consistent with findings from Cetin et al. (48.8%) (33), but higher than in other cohorts (37).

Infection with hrHPV was observed in 100% of the study population. Moreover, simultaneous infection with hrHPV and lrHPV was present in 17.1% of cytology samples from patients, which is higher than that reported in other studies (38–40). This disparity is because our inclusion criteria were patients with hrHPV, and Kowsar Hospital is a referral hospital. Sundström et al. demonstrated that coinfection with lrHPV is associated with a decreased likelihood of developing subsequent invasive disease and a prolonged period before diagnosis compared with infection with hrHPV alone (39).

Based on colposcopy findings, acetowhite lesions were the most frequent (45.6%). In addition, the results of colposcopy in 21.2% of the patients were normal. Similarly, Zhou et al. showed that the visual appearance of the uterine cervix varies depending on the HPV type status in cases of high-grade squamous intraepithelial lesions, and that the characteristic acetowhite epithelium is primarily associated with lesions positive for HPV16, which is an hrHPV (41). In contrast, a significant correlation was observed between colposcopy findings and smoking. Studies have shown that the likelihood of HPV infection increases twofold in women who smoke compared to those who do not. Additionally, smoking is linked to higher rates of cervical abnormalities detected through both colposcopy and cytology (42,43). The higher rate of HPV persistence in smokers is associated with several factors related to immune system suppression caused by smoking. Smoking reduces the number of Langerhans cells in the cervical epithelium, which are crucial for detecting and presenting viral antigens to the immune system. Additionally, smoking affects T cell function by increasing CD8+ cells and decreasing CD4+ cells, thereby impairing the body's ability to fight infections. Moreover, in smokers there is weakened natural killer cell activity and higher viral loads of HPV types 16 and 18, all of which contribute to persistent infections (44).

According to the pathological findings, CIN1 was the most frequent (72.4%). Our results are higher than those reported in other studies. For example, Cetin et al. found that punch biopsy results showed CIN1 in 48.4% of HPV-positive patients, CIN2 or CIN3 in 8.1%, squamous cell carcinoma in 1.1%, and cervicitis in 30.4% of the patients (33). However, in a study conducted by Rad et al. on 1000 women with abnormal Pap smears, CIN1 was the most common condition, occurring in 64.4% of cases, followed by cervicitis in 29.1% of patients (20). Briolat et al. showed that the prevalence of HPV16 increased with lesion severity, with 27.1% in CIN1 and 65.3% in CIN3. Additionally, the frequency of single infections, compared to multiple infections, increased with lesion severity from 25.0% in CIN1 to 54.8% in CIN3 (45).

In a study by Ding et al. on 240 patients with cervical squamous intraepithelial lesions, it was shown that the prevalence of HSIL was greatest in patients aged 31–40. They proposed that colposcopy should be performed to detect cervical lesions early and initiate treatment without delay, regardless of the presence of hrHPV infection (46). In our study of hrHPV patients, the highest frequency was seen in patients with ASCUS, followed by LSIL, in the 30–39 age group.

In a study by Spinillo et al. involving 2985 women diagnosed with either CIN or cancer, CIN3 diagnoses were more common among those co-infected with HPV16 and HPV18 or HPV52 compared to those with only HPV16 infection. Additionally, multiple infections did not affect residual disease or recurrence of high-grade CIN. HPV16 showed positive interaction with HPV18 and negative interaction with HPV33, 51, 52, and 66, supporting the idea that HPV16 generally interacts negatively with other hrHPVs in CIN lesions (47).

In a study by Liu et al. involving 3,134 patients with HPV infection, 55.68% and 43.20% of the patients had HSIL and LSIL, respectively, with the highest incidence observed among women aged 35–49 years. The rates of single-genotype HPV infection and multiple-genotype infections were 55.26% and 34.18%, respectively. There was no significant difference in the proportion of multiple genotype infections among those with HSIL, LSIL, and normal results, and the presence of multiple HPV genotypes did not increase the risk of HSIL. The prevalence of specific HPV types and their contribution to cervical precancerous lesions should be considered when developing screening strategies for this population (48).

Sung et al. found an 11.1% prevalence of hrHPV infection and recommended co-testing with HPV assay and liquid-based cytology for cervical cancer screening. They emphasized the importance of detecting "genotypes excluding HPV16/18" separately in screening strategies. The prevalence of hrHPV and HPV16 increases with increasing cytological severity. For HPV16-positive women with normal cytology, the baseline risk of CIN2/3 or worse is approximately 20% (40). Moreover, Ribbe et al. demonstrated that clinical outcomes vary significantly with different HPV genotypes, and high-risk types like HPV16 and HPV18 are more closely associated with CIN3 and cancer (49). Based on these studies, screening strategies must account for genotype-specific risks, with more frequent monitoring for those infected with hrHPV types.

In this study, the sensitivity (69.1%), specificity (42.5%), and positive predictive value (74.9%) of colposcopy were comparable to those reported in similar studies of HPV-positive patients (50). The high sensitivity aligns with previous research, confirming the diagnostic accuracy of the method for detecting abnormal cervical lesions, despite the modest specificity, which could increase biopsy referrals if colposcopy is used alone. Most studies report lower specificity for colposcopy compared to Pap smears, suggesting that colposcopy should be performed alongside Pap smears to detect cervical lesions effectively (20,51).

The sensitivity (47.9 %), specificity (63.1 %), and positive predictive value (76.2 %) of the pap smear were consistent with other studies, which reported a sensitivity of 55.5%, specificity of 75%, and positive predictive value of 88.2% (52). Despite its low sensitivity, the pap smear has a high positive predictive value, accurately identifying true positive cases but fails to distinguish between high and low grades of atypia. Therefore, additional methods must be used to align with pap smears to improve the diagnosis.

The limitations of this study include that it was a single-center design at Kowsar Qazvin Hospital, which restricts the generalizability of the findings and the demographic biases in the patient population that do not reflect national averages. The limited data on other parameters, such as socioeconomic status and sexual behavior, may have an important influence on hrHPV infection and cervical lesion development. Additionally, comparing hrHPV patients with those without HPV could provide valuable insights into the effectiveness of different diagnostic methods in these two populations. Future research should involve multicenter studies to enhance generalizability, comprehensive data collection including socioeconomic and behavioral factors, utilizing advanced diagnostic tools, and focusing on effective preventive strategies like targeted HPV vaccination and screening programs are essential future directions. Moreover, future research should explore the relationship between individual hrHPV types and the severity of cervical lesions to understand their contribution to disease progression better.

## Conclusion

This study provides significant insights into the prevalence and clinical characteristics of multiple hrHPV infections among women at a referral hospital in Iran, highlighting a higher incidence in women aged 30–39 and the critical need for early detection and intervention. Despite its limitations, the study emphasizes the importance of combining Pap smear and colposcopy for accurate diagnosis. These findings underscore the necessity for comprehensive screening strategies and the importance of addressing identified limitations in future research to enhance understanding and develop effective preventive and therapeutic measures for hrHPV infections. Moreover, to reduce the burden of HPV-related cervical cancer, it is crucial to enhance vaccination drives and public awareness campaigns, particularly in high-risk populations, to promote early detection and prevention.
